# Medicine, Volume 95, Issue 23: Erratum

**DOI:** 10.1097/01.md.0000489580.04709.16

**Published:** 2016-07-18

In volume 95, issue 23 of Medicine, a large number of article PDFs published with an incorrect online publication date of May 1, 2016. That date has since been removed from the articles in question.
